# Effectiveness of Microwave Ablation for the Treatment of Epistaxis: A Systematic Review and Meta-Analysis

**DOI:** 10.7759/cureus.86270

**Published:** 2025-06-18

**Authors:** Kenny H Do, Eric Kawana, Kurtis Young, Sisi Tian, Jee-Hong Kim, Jo-Lawrence Bigcas

**Affiliations:** 1 Department of Otolaryngology, Head and Neck Surgery, Kirk Kerkorian School of Medicine at the University of Nevada, Las Vegas (UNLV), Las Vegas, USA

**Keywords:** epistaxis, microwave ablation, minimally invasive procedure, nosebleeds, rhinology

## Abstract

Epistaxis is a common condition that is often benign and does not require serious intervention. The use of microwave ablation (MWA) is a relatively new technique for treating epistaxis. This systematic review examines the efficacy and safety of using MWA to treat common nosebleeds. It was conducted in accordance with the Preferred Reporting Items for Systematic Reviews and Meta-Analyses (PRISMA). Two databases, PubMed and EMBASE, were used to identify and aggregate articles for this systematic review. The search terms yielded a total of 2,691 articles with years ranging from 1947 to 2023. After screening, one case series, one randomized control trial, and five observational studies fit the inclusion criteria. Minimal complications were experienced by patients who underwent MWA treatment.

A total of 876 patients underwent MWA, with 852 reporting no recurrence at 3 or 6 months, indicating a 97.3% success rate. Three of the seven studies were pooled in a meta-analysis, revealing a pooled log odds ratio of 2.05 (95% CI: 1.19-2.91; p = 0.00), indicating higher odds of recurrent bleeding with observation or silver nitrate versus MWA. In a study of 83 patients, the average pain score was 1.83 during the MWA procedure and decreased to 0.95 one hour afterward. Overall, MWA is a safe and effective treatment for epistaxis, with low rebleeding and complication rates. Further research and a meta-analysis comparing MWA with traditional treatments are recommended to enhance our understanding.

## Introduction and background

Epistaxis is a common condition experienced by approximately 60% of the population worldwide, where only 6-10% of these cases require immediate medical treatment [1,2]. Typically caused by ruptured nasal mucosa vessels, epistaxis may spontaneously occur or result from trauma, systemic diseases (i.e. sinus tumors, juvenile nasal angiofibroma, hereditary hemorrhagic telangiectasia, and granulomatous diseases), or as side effects of medications like anticoagulants, with contributing factors being mucosal dryness, allergic rhinitis, hypertension, and diabetes [3].

The most common site for nosebleeds to occur is at Kiesselbach’s plexus located in the anterior septum of the nose [4]. The tributaries of the anterior ethmoid, greater palatine, sphenopalatine, and superior labial arteries comprise this plexus [3,4]. Additionally, posterior epistaxis can result in bleeding at the rear of the nasal cavity, typically occurring at Woodruff’s plexus, which comprises the pharyngeal and sphenopalatine arteries [4,5].

Treatment options for epistaxis range from conservative measures like nasal packing and topical vasoconstrictors to more invasive procedures such as silver nitrate cauterization, endoscopic clipping, and percutaneous embolization [1,6]. However, these approaches have notable drawbacks, including patient discomfort, high recurrence rates, and potential complications. Nasal packing is often painful and poorly tolerated, causing obstructed breathing and a reduced sense of smell. Silver nitrate cauterization may be ineffective for posterior or diffuse bleeding. Although embolization is effective, it carries serious risks, such as tissue necrosis and stroke, and is not universally available [1,6].

Microwave ablation (MWA) is another procedure that can be used to treat epistaxis, where this technique has been widely implemented in the medical community for intraoperative and postoperative hemostasis [7]. MWA uses intense heat and energy generated from microwaves to cause coagulative necrosis of the targeted tissue [8,9]. This promotes the destruction of tumors or coagulation of blood vessels to maintain hemostasis, where temperatures typically go over 50 °C [9,10]. Previous studies have demonstrated the safe use of MWA in the liver, pancreas, endometrium, thyroid, and more [11-14]. While MWA has been successfully used for intraoperative and postoperative hemostasis in organs like the liver and thyroid, its application in treating recurrent or uncontrolled nosebleeds is recent. In accordance with the Patient/Problem, Intervention, Comparison, Outcome (PICO) framework, the following question was created for this systematic review: in adult and pediatric patients with epistaxis, how does MWA affect rebleeding rates, pain scores, and complications following treatment? The authors hypothesize that MWA will be effective and safe in treating epistaxis that has failed traditional treatment modalities, serving as a viable alternative for treating refractory epistaxis.

## Review

Methods

Study Design

**Figure 1:**
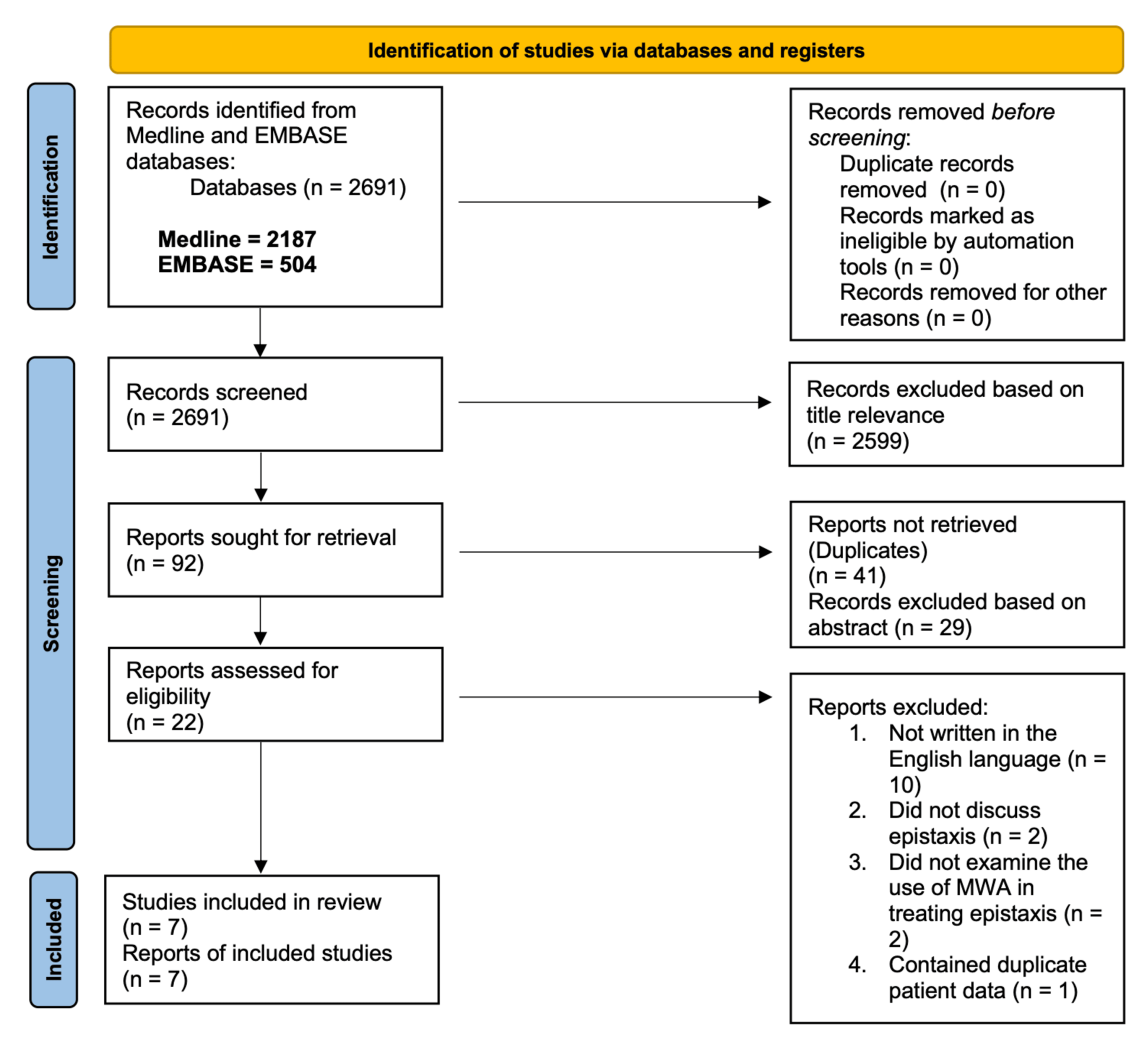
Study flow chart

Two comprehensive medical databases, PubMed (MEDLINE) and Excerpta Medica (EMBASE), were used to search articles for this systematic review. The method of this study relies on the Preferred Reporting Items for Systematic Reviews and Meta-Analyses (PRISMA) systematic review guideline [15]. Two independent reviewers (KD and EK) employed the search terms “Epistaxis Manage” and “Epistaxis Microwave” in the two databases. Using Boolean operators, such as “AND” and “OR”, additional search terms in Table 1 were entered in PubMed and Embase. The search on PubMed yielded 2187 results (1947-2023), and the search on EMBASE yielded 504 results (1975-2023). The five steps involved in the screening of these articles are demonstrated in Figure 1. Inclusion criteria for this systematic review involved papers that examined the use of MWA in treating acute or recurring epistaxis. The exclusion criteria for this systematic review encompassed studies that did not focus on epistaxis, did not investigate the application of MWA in treating epistaxis, were not written in English, were not published as complete full-length papers, or contained duplicate patient data. The risk of bias for the studies included in this systematic review was evaluated in accordance with the National Institute of Health (NIH) Study Quality Assessment tool [16]. This study has not been registered in PROSPERO, and data are available upon request.

**Table 1:** Additional search terms on PubMed and Embase

Database	Search Terms
PubMed	("Epistaxis"[Mesh] or epistaxis or nosebleed or nose bleed or nose bleeding or nasal hemorrhage or nasal bleed or nasal bleeding) AND ("Microwaves/therapeutic use"[Mesh] or microwave ablation or microwave or MWA)
Embase	('epistaxis' OR 'epistaxis'/exp OR epistaxis OR 'nosebleed'/exp OR nosebleed OR 'nose bleed'/exp OR 'nose bleed' OR (('nose'/exp OR nose) AND bleed) OR 'nose bleeding'/exp OR 'nose bleeding' OR (('nose'/exp OR nose) AND ('bleeding'/exp OR bleeding)) OR 'nasal hemorrhage'/exp OR 'nasal hemorrhage' OR (nasal AND ('hemorrhage'/exp OR hemorrhage)) OR 'nasal bleed' OR (nasal AND bleed) OR 'nasal bleeding'/exp OR 'nasal bleeding' OR (nasal AND ('bleeding'/exp OR bleeding))) AND ('microwaves/therapeutic use' OR 'microwave ablation'/exp OR 'microwave ablation' OR (('microwave'/exp OR microwave) AND ablation) OR 'microwave'/exp OR microwave OR mwa)

Study Selection

Two independent reviewers (KD and EK) screened articles, with a senior researcher (JLB) resolving any disagreements. Initially, broad search terms were applied in PubMed and EMBASE, then article titles were reviewed for relevance. Next, abstracts discussing MWA in epistaxis treatment were examined, leading to a full-text review. Eligible articles included case series, observational studies, and randomized controlled trials. Exclusions were made for articles not focused on MWA in epistaxis, lacking peer review, or not in English. Figure 1 details the selection process, resulting in 7 articles making it through screening.

Quality Assessment and Data Abstraction

The risk of bias for the observational studies, randomized control trials, and case series included in this systematic review was evaluated in accordance with the National Institute of Health (NIH) Study Quality Assessment tool [16]. The individual observational studies and randomized control trials were evaluated based on a 14-question questionnaire. Scores ranging from 11-14 were considered good quality, 7-10 were considered fair quality, and 0-6 were considered poor quality [16]. The risk of bias for case series was assessed using a nine-question questionnaire. Scores ranging from 7-9 were considered good quality, 5-6 were considered fair quality, and 0-4 were considered poor quality [16].

Data Analysis

SPSS software version 29 (IBM Corp., Armonk, New York, USA) was used to calculate the meta-analysis across three papers in this study. A random effects model utilizing restricted maximum likelihood (REML) estimation was used to determine the logarithmic odds ratios for recurrent bleeding among different epistaxis treatment methods [17,18]. These odds ratios, in their logarithmic form, were displayed in a forest plot. Heterogeneity was evaluated with the I2 index, where values of 0-50% indicated low heterogeneity, 50-75% indicated moderate heterogeneity, and 75-100% indicated high heterogeneity [19]. The level of significance (α) was set at 0.05 as a reference [20].

Results

In accordance with the PRISMA guidelines, seven articles were identified for this systematic review, which included five observational studies, one randomized controlled study, and one case series. These studies analyzed the efficacy, adverse effects, and recurrence of bleeding in patients undergoing MWA for the management of their epistaxis. Some articles also investigated comparisons between MWA and traditional epistaxis treatment options.

Study Screening

The PRISMA protocol began with assessing titles from PubMed and EMBASE, identifying 92 relevant papers. The removal of 41 duplicates left 51, whose abstracts were reviewed, discarding 29. Full-text evaluation of the remainder included seven studies in this review. Exclusions were due to a lack of focus on MWA for epistaxis, language, or incomplete publication status. One paper was removed because it contained duplicate patient data.

Study Quality

The National Institute of Health (NIH) Study Quality Assessment tool [16] was used to evaluate the quality and risk of bias of the seven individual studies included in this systematic review. After examination by two independent reviewers, it was found that six of the papers were of fair quality and one was of good quality, which are highlighted in Tables 2-4.

**Table 2:** Risk of bias assessment for case series Study Quality Assessment Tools | National Health Lung and Blood Institute (NHLBI), National Institutes of Health (NIH). Accessed March 21, 2023. https://www.nhlbi.nih.gov/health-topics/study-quality-assessment-tools [16]

Criteria	Lou 2019 [21]
Q1: Was the study question or objective clearly stated?	Y
Q2: Was the study population clearly and fully described, including a case definition?	Y
Q3: Were the cases consecutive?	N
Q4: Were the subjects comparable?	N
Q5: Was the intervention clearly described?	Y
Q6: Were the outcome measures clearly defined, valid, reliable, and implemented consistently across all study participants?	Y
Q7: Was the length of follow-up adequate?	Y
Q8: Were the statistical methods well-described?	N
Q9: Were the results well-described?	Y
Final Quality Score	6
Rating	Fair

**Table 3:** Risk of bias assessment for controlled intervention studies Study Quality Assessment Tools | National Health Lung and Blood Institute (NHLBI), National Institutes of Health (NIH). Accessed March 21, 2023. https://www.nhlbi.nih.gov/health-topics/study-quality-assessment-tools [16]

Criteria	Lou 2019 [22]
Q1: Was the study described as randomized, a randomized trial, a randomized clinical trial, or an RCT?	Y
Q2: Was the method of randomization adequate?	Y
Q3: Was the treatment allocation concealed?	Y
Q4: Were the study participants and providers blinded to treatment group assignment?	N
Q5: Were the people assessing the outcomes blinded to the participants’ group assignments?	N
Q6: Were the groups similar at baseline on important characteristics that could affect outcomes?	Y
Q7: Was the overall drop-out rate from the study at endpoint 20% or lower of the number allocated to treatment?	Y
Q8: Was the differential drop-out rate (between treatment groups) at endpoint 15 percentage points or lower?	Y
Q9: Was there high adherence to the intervention protocols for each treatment group?	Y
Q10: Were other interventions avoided or similar in the groups (e.g., similar background treatments)?	Y
Q11: Were outcomes assessed using valid and reliable measures, implemented consistently across all study participants?	Y
Q12: Did the authors report that the sample size was sufficiently large to be able to detect a difference in the main outcome between groups with at least 80% power?	N
Q13: Were outcomes reported or subgroups analyzed prespecified?	Y
Q14: Were all randomized participants analyzed in the group to which they were originally assigned?	Y
Final Quality Score	11
Rating	Good

**Table 4:** Risk of bias assessment for observational studies Study Quality Assessment Tools | National Health Lung and Blood Institute (NHLBI), National Institutes of Health (NIH). Accessed March 21, 2023. https://www.nhlbi.nih.gov/health-topics/study-quality-assessment-tools [16]

Criteria	Lou 2019 [23]	Lou et al, 2019 [24]	Lou 2020 [25]	Lou 2021 [26]	Lou et al, 2019 [27]
Q1: Was the research question or objective in this paper clearly stated?	Y	Y	Y	Y	Y
Q2: Was the study population clearly specified and defined?	Y	Y	Y	Y	Y
Q3: Was the participation rate of eligible persons at least 50%?	Y	Y	Y	Y	Y
Q4: Were all the subjects selected or recruited from the same or similar populations (including the same time period)? Were inclusion and exclusion criteria for being in the study prespecified and applied uniformly to all participants?	Y	Y	Y	Y	Y
Q5: Was a sample size justification, power description, or variance and effect estimates provided?	N	N	N	N	N
Q6: For the analyses in this paper, were the exposure(s) of interest measured prior to the outcome(s) being measured?	Y	Y	Y	Y	Y
Q7: Was the timeframe sufficient so that one could reasonably expect to see an association between exposure and outcome if it existed?	Y	Y	Y	Y	Y
Q8: For exposures that can vary in amount or level, did the study examine different levels of the exposure as related to the outcome (e.g., categories of exposure, or exposure measured as continuous variable)?	N	N	N	N	N
Q9: Were the exposure measures (independent variables) clearly defined, valid, reliable, and implemented consistently across all study participants?	Y	Y	Y	Y	Y
Q10: Was the exposure(s) assessed more than once over time?	N	Y	Y	Y	Y
Q11: Were the outcome measures (dependent variables) clearly defined, valid, reliable, and implemented consistently across all study participants?	Y	Y	Y	Y	Y
Q12: Were the outcome assessors blinded to the exposure status of participants?	N	N	N	N	N
Q13: Was loss to follow-up after baseline 20% or less?	Y	Y	Y	Y	Y
Q14: Were key potential confounding variables measured and adjusted statistically for their impact on the relationship between exposure(s) and outcome(s)?	N	N	N	N	N
Final Quality Score	9	10	10	10	10
Rating	Fair	Fair	Fair	Fair	Fair

Study Characteristics

The review covered 876 patients (average age: 50.1 years for adults, 15.3 for children; 589 male, 296 female) across 7 studies. Initially, 885 were enrolled; exclusions were due to fear of MWA (7 children)21 or loss to follow-up (2 adults)22. Among the analyzed, 852 showed no recurrence at 3- or 6-month checks, affirming a 97.3% success rate. Detailed outcomes are in Table 5.

**Table 5:** Epistaxis patients treated with microwave ablation (MWA) ^1^Risk of Bias Assessment for Case Series ^2^Risk of Bias Assessment for Controlled Intervention Studies ^3^Risk of Bias Assessment for Observational Studies

Article	Study Type	Mode	Number of patients treated with MWA.	Number of MWA Patients with no Rebleeding	Total Percent with no rebleeding	MWA Efficacy	Keen findings	Risk of Bias Assessment
Lou, 2019 [21]	Prospective Case Series	50 W	85	85	100	None of the patients who had MWA had recurrent bleeding. 92 patients were initially enrolled for MWA treatment. However, 7 patients did not have complete MWA due to fear and were later treated with silver nitrate cautery.	No serious complications were observed at six months.	Fair^1^
Lou et al, 2019 [22]	Prospective, Randomized Controlled Study	50 W	48	46	95.8	Initially, 50 patients were enrolled in MWA treatment. At 6 months, 2 patients were lost to follow-up, and 2 had recurrent bleeding, compared to the silver nitrate cautery group, where 7 were lost to follow-up and 17 had recurrent bleeding.	11 patients in the MWA group had some pain after treatment compared to 50 in the silver nitrate cautery group. No severe complications were observed at 6 months.	Good^2^
Lou 2019 [23]	Retrospective Observational Study	60 W	39	32	82.1	39 patients received prophylactic microwave ablation, where at 3 months, rebleeding was observed in 7 of these patients. In the control group with only observation, 13 out of 22 patients experienced rebleeding.	Microwave ablation was effective after 10-20 seconds in all patients, where 11 patients had ablation to one area of the nasal cavity, 23 patients had ablation to 2 areas, and 5 patients had ablation to 3 areas.	Fair^3^
Lou et al, 2019 [24]	Retrospective Observational Study	40-60W	71	71	100	Bleeding points were identified and ablated in 67 patients, while 4 patients received prophylactic ablation. No recurrent bleeding in any of the patients.	For the 67 patients with posterior epistaxis, 44 had bleeding sites at the olfactory cleft, 5 at the middle meatus, and 18 at the inferior meatus. 19 patients had minor errhysis after the procedure.	Fair^3^
Lou 2020 [25]	Retrospective Observational Study	60 W	69 (at 3 and 6 months)	66 (at 3 months), 68 (at 6 months)	95.7 (at 3 months) and 98.6 (at 6 months)	At 3 months, 3 patients had recurrent bleeding, while 6 in the silver nitrate cautery group had recurrent bleeding. At 6 months, 1 patient had recurrent bleeding while 4 in the silver nitrate cautery group had recurrent bleeding.	At 3 months, no serious complications following the MWA procedure. At 6 months, no serious complications following the procedure. Crusting was observed in 7 (10.14%) in the MWA group, which was significantly less than 47 (58.02%) in the silver nitrate cautery group.	Fair^3^
Lou 2021 [26]	Retrospective Observational Study	60-80W	83	83	100	83 patients had bleeding points on unilateral isolated mucosal bulge lesions. At the 6-month follow-up, no rebleeding was seen.	18% of patients experienced crusting after 2 weeks. No other severe complications.	Fair^3^
Lou et al, 2019 [27]	Retrospective observational Study	60 W	481	469	97.5	At 6 months, 469 patients were followed up with no recurrent bleeding and no severe complications.	167 (34.7%) had minor pain right after the procedure. 139 (28.9%) had rhinorrhea 1 week after the procedure.	Fair^3^

Recurrent Bleeding

The efficacy of MWA in treating epistaxis can be assessed by analyzing the recurrence rate among patients. Across the seven studies, the follow-up time of either three or six months was chosen to assess the number of recurrent bleedings in patients treated with MWA in this systematic review [21-27]. Some of the individual articles only specified rebleeding at three months, and some only specified rebleeding at six months.

Lou (2019) [23], Lou et al. (2019) [24], and Lou (2020) [25] examined the efficacy and safety of using MWA to treat idiopathic recurrent anterior epistaxis and posterior epistaxis. At 3 months, Lou (2019) [23] reported 32 out of 39 patients (82.0%) who did not experience rebleeding following MWA treatment. Lou et al. (2019) [24] identified and ablated bleeding points in 67 patients, as well as providing prophylactic ablation in four patients with posterior epistaxis. None of the 71 patients experienced rebleeding at the three-month follow-up. Lou (2020) [25] studied patients with idiopathic recurrent anterior epistaxis, and at the 3-month follow-up, 66 out of 69 (95.7%) patients did not experience recurrent bleeding.

Some of the studies [21,22,25,27] reported the rate of recurrent bleeding at the six-month follow-up. Lou (2021) [26] reported that none of the 83 patients with unilateral isolated mucosal bulge lesions in this study had recurrent bleeding after MWA treatment. Another study by Lou et al. (2019) [27] retrospectively observed 481 patients and found that at the 6-month follow-up, 469 (97.5%) of patients did not have recurrent bleeding. Lou (2020) [25] studied that at 6 months, only 1 patient had recurrent bleeding, meaning 68 out of 69 (98.6%) were successfully treated with MWA. Pediatric and adolescent patients with idiopathic recurrent epistaxis were also treated with MWA in Lou (2019) [21], where all 85 patients who underwent MWA had no recurrent bleeding. In this study, 7 out of the initial 92 patients refused MWA treatment due to their being fearful of the equipment. We did not include these 7 patients in the calculations. Lou et al. (2019) [22] reported that out of the 50 patients, 2 patients were lost to follow-up. As a result, out of the 48 remaining patients who underwent MWA, 2 had recurrent bleeding at 6-month follow-up, yielding an efficacy rate of 95.3% [22].

Pain Scores

The visual analog scale (VAS) is a tool utilized for pain assessment, featuring a scale from 0 to 100, with higher scores on the scale corresponding to heightened levels of pain [28]. One study consisting of 83 patients reported a mean pain score of 1.83 during the MWA procedure and 0.95 one hour following the procedure [26].

Comparison to Other Modalities

Three out of the seven articles included in this systematic review compared the use of MWA to other treatment options, such as only observation or silver nitrate cautery. Lou (2019) [23] studied 39 patients in the MWA group and 22 patients in the observation-only group. At the 3-month follow-up, there were 7 cases of recurrent bleeding in patients who received MWA versus 13 in the observation group [23].

In another study, patients with idiopathic recurrent anterior epistaxis were treated with MWA or silver nitrate cautery [25]. At three-month follow-up, three cases of rebleeding were reported in the MWA group, whereas six cases were reported in the silver nitrate cautery group. At the six-month follow-up, one case of rebleeding was reported in the MWA group, whereas four cases were reported in the silver nitrate cautery group. Furthermore, crusting was seen in 10.14% of the MWA patients versus 58.02% in the silver nitrate cauterization patients [25].

Lou et al. (2019) [22] compared the use of MWA and silver nitrate cautery in adolescent patients. One hundred patients were included in this study, with 50 in the MWA group and 50 in the silver nitrate cauterization group. At six-month follow-up, two patients had rebleeding, and two patients were lost to follow-up in the MWA group. In the silver nitrate cauterization group, 17 patients had rebleeding, and 7 were lost to follow-up. Furthermore, 11 patients in the MWA group reported minor pain compared to the 50 patients in the silver nitrate cauterization group who reported minor pain [22].

Meta-Analysis: Comparison to Other Modalities for Recurrent Bleeding

**Figure 2:**
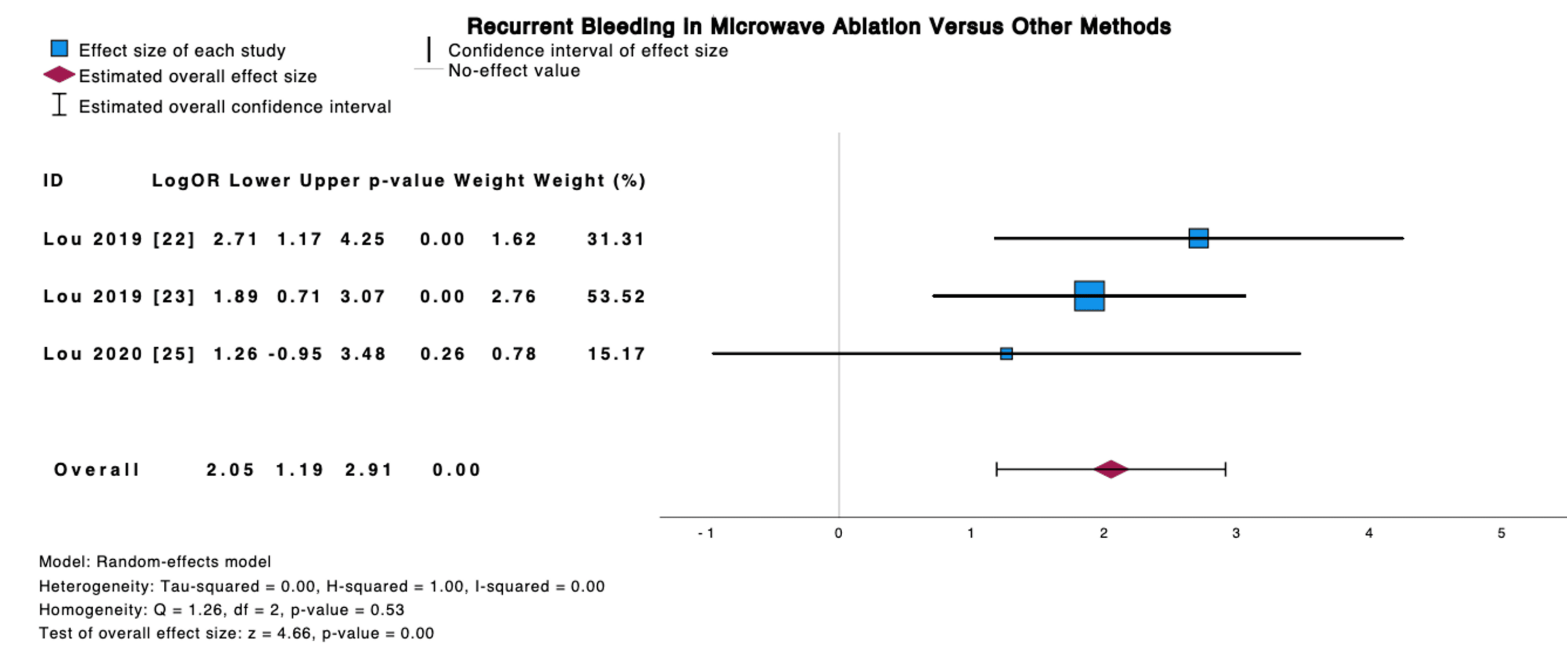
Logarithmic odds ratio for recurrent bleeding in MWA vs other methods Source: [22,23,25] MWA: microwave ablation

The pooled logarithmic odds ratio in Figure 2 for Lou (2019) [23], Lou (2020) [25], and Lou et al. (2019) [22] demonstrated higher odds ratio for recurrent bleedings in either observation only or silver nitrate groups when compared to the MWA group (2.05 (95%CI: 1.19 to 2.91, p-value: 0.00). Heterogeneity was low, with the I2 index being 0.00.

Complications

Few and minor complications were reported in the seven studies included in this systematic review. For example, one study reported that out of 83 patients, 18% of patients experienced yellow crusting 2 weeks following MWA treatment [26]. Another study reported that out of 481 patients, 34.7% experienced minor pain in their nose and 28.9% reported rhinorrhea one week following the procedure [27]. Additional research conducted by this physician revealed no significant or life-threatening adverse effects following MWA treatment, including complications such as septal perforation, synechiae formation, or issues involving the orbit and brain.

Discussion

MWA has been widely used for hemostatic control in various settings, including the thyroid gland, liver, and spleen [6,7,11,12]. This minimally invasive procedure employs an electromagnetic field to achieve temperatures typically ranging from 65 °C to 100 °C, with the potential to exceed 150 °C within tissues [22,29]. The use of MWA first started in 1994, and advancements in technology have allowed it to serve as an alternative to radiofrequency ablation (RFA). Offering advantages over RFA, including faster temperature rise and a higher temperature limit, MWA allows for quicker ablation times [29,30]. Operating at 915-2450 MHz, versus RFA's 450-500 kHz, MWA produces larger coagulation zones and is more effective in moist tissues, reducing heat loss in vascular areas [29-31].

This systematic review compares MWA with two other epistaxis treatment modalities: silver nitrate cauterization and electrocoagulation, both commonly employed to achieve hemostasis. In the literature, one study reported that electrocoagulation had a recurrence rate of 2% compared to 18% in chemical coagulation [32]. Articles in this review suggest MWA has lower recurrence rates than both observation alone and silver nitrate cauterization, as shown in the Results section [22,23,25]. Moreover, the attributes of MWA highlighted earlier, such as its capacity to coagulate larger surface areas and precise temperature control, enable it to achieve quicker hemostasis compared to other techniques [29,33,34]. Additionally, its versatility extends to both tumor destruction and hemostasis, unlike chemical cauterization or electrocoagulation, which are limited to hemostasis alone [33]. This review shows that MWA-treated patients experienced fewer complications, like postoperative pain, rhinorrhea, and crusting, than those treated with electrocoagulation or silver nitrate cauterization.

Other methods are available in treating epistaxis as well [6,35,36]. Nasal packing uses materials such as specialized gauze or sponges to apply constant pressure in the bleeding vessels [35,36]. One study examining 969 patients found that nasal packing had an efficacy rate of 87.5%, with there being a positive correlation between the duration of the nasal packing and the success of hemostasis [35]. Tranexamic acid (TXA) can be used to stop uncontrolled epistaxis as well [36]. One study comparing the efficacy of TXA versus nasal packing reported that in 216 patients, 71% of patients who received TXA achieved hemostasis within 10 minutes, while only 31.2% of patients who received anterior nasal packing achieved hemostasis within 10 minutes [36,37]. Furthermore, within the first 24 hours, 4.7% of TXA patients had recurrent bleeding compared to 12.8% of patients in the nasal packing group [36,37]. Endoscopic surgical ligation of the sphenopalatine artery can be performed as well, with efficacy rates ranging from 92% to 100% [6]. By examining the efficacy rates of these techniques against those of MWA, the existing literature suggests that MWA can match the performance of some conventional methods in treating recurrent epistaxis, while also offering potential advantages. Additional studies, particularly randomized controlled trials, are needed to directly compare the success rates of MWA with those of other techniques.

Cost is another important consideration. Lou reported that in his studies, the average cost of silver nitrate cautery was approximately $3.70, and the average cost of bipolar electrocautery was approximately $25 [25,33]. Lou also stated that in his country, the cost of RFA can be as high as $500 per treatment [33]. The average cost for MWA was approximately $13 [17,33]. While chemical cauterization proves to be less expensive for patients in the study, the author noted that because of the requirement for repeated cauterizations, MWA ultimately emerges as a more cost-effective option when compared to other options [25]. However, it is essential to recognize that the cost of using MWA varies between countries. To our knowledge, MWA has not been utilized in the United States for treating epistaxis, which makes it challenging to determine its cost. Consequently, it is possible that using MWA in the U.S. could be significantly more expensive than other methods. More studies are needed to understand the cost analysis of chemical cauterization, RFA, MWA, and other modalities in different parts of the world.

Recurrence rates were low across the 7 studies in this review, ranging from 0% to 17.9% [24,26]. The studies also showed MWA's efficacy in both pediatric and adult patients, with 133 pediatric and 795 adult patients treated, showing similar success rates. Recurrences were rare in both groups. Pediatric patients primarily faced issues with follow-up loss or apprehension towards MWA equipment [21].

Limitations

A significant limitation is the homogeneity of sources, with all studies coming from the same authors, raising the possibility of bias. Despite verification that all subjects were unique, the lack of diverse authorship underscores the need for more varied research, including randomized controlled trials, to further validate MWA's effectiveness in treating epistaxis. The purpose of this review is not to promote the work of a single author but to aggregate and critically examine all the available literature on microwave ablation for epistaxis. These seven articles are the only literature available on this topic. Furthermore, the exclusion of non-English studies and gray literature represents a potential source of language and publication bias. This may have limited the comprehensiveness of the evidence base and could affect the generalizability of the findings.

It is important to note that we had contacted the corresponding author of these seven articles and confirmed that all the MWA patients in these studies are unique individuals and not repeat participants. There are no conflicts of interest between the investigators of this systematic review and the author(s) of the individual studies chosen for this paper.

Future directions may include the incorporation of a meta-analysis into our data. It would be useful to conduct a meta-analysis that directly compares the efficacy rate of common epistaxis treatment options, such as nasal packing, hemostatic gauze, electrocauterization, chemical coagulation, and surgery, to that of MWA.

## Conclusions

Microwave ablation (MWA) presents an effective and affordable alternative for treating epistaxis, a widely prevalent condition. This review confirms MWA's efficacy, showcasing a notably low rate of rebleeding for recurrent or idiopathic cases, with minimal patient complications. Moreover, the evidence suggests MWA's effectiveness could equal or surpass traditional methods like chemical cauterization or electrocautery. More studies, especially randomized controlled studies, are needed to better understand the use of MWA in treating epistaxis.
